# Spot specimen testing with GeneXpert MTB/RIF results compared to morning specimen in a programmatic setting in Cotonou, Benin

**DOI:** 10.1186/s12879-021-06676-6

**Published:** 2021-09-20

**Authors:** Faridath Massou, Merlin Fandohan, Ablo Prudence Wachinou, Schadrac Christin Agbla, Gildas Agodokpessi, Leen Rigouts, Bouke Catherine de Jong, Dissou Affolabi

**Affiliations:** 1 National Tuberculosis Program, NTP, Cotonou, Benin; 2 Supranational Reference Laboratory for Tuberculosis of Cotonou, Cotonou, Benin; 3 London School of Hygien and Tropical Medicine, London, UK; 4 University of Liverpool, Liverpool, UK; 5 Institute of Tropical Medicine, Antwerp, Belgium

**Keywords:** GeneXpert® MTB/RIF, Screening strategy, Specimens

## Abstract

**Background:**

The diagnosis of tuberculosis (TB) using smear microscopy has been based on testing two specimens: one spot and one early morning sputa. Recently, the World Health Organization (WHO) has recommended to replace, whenever possible, microscopy with GeneXpert® MTB/RIF performed on a single specimen. However, as the bacterial load is higher in early morning specimens than in spot specimens, one could expect lower sensitivity of GeneXpert® MTB/RIF performed only on spot specimens. In this study, we compared results of GeneXpert® MTB/RIF on spot specimens versus early morning specimens, under programmatic conditions in Cotonou, Benin.

**Methods:**

From June to September 2018, all sputa received from presumptive TB patients at the Supranational Reference Laboratory for Tuberculosis of Cotonou were included in the study. From each patient, two specimens were collected (one spot and one early morning) and GeneXpert® MTB/RIF was performed on both specimens.

**Results:**

In total, 886 participants were included in the study, of whom 737 provided both sputa and 149 (16.8%) gave only the spot specimen. For the 737 participants who provided both sputa, GeneXpert® MTB/RIF was positive for both specimens in 152 participants; for three participants GeneXpert® MTB/RIF was positive on spot specimen but negative on morning specimen while for another three, the test was positive on morning specimen but negative on spot specimen. The overall percentage of agreement was excellent (99.2%) with a positive and negative percent agreement greater than 98%.

**Conclusion:**

For TB diagnosis under programmatic conditions in Cotonou, GeneXpert® MTB/RIF in spot specimens gave similar results with the test in morning specimens. Performing GeneXpert® MTB/RIF in both specimens did not significantly increase the number of cases detected. To avoid losing patients from the diagnostic cascade, it is preferable to test sputa produced at the time of the first visit at the health center.

**Supplementary Information:**

The online version contains supplementary material available at 10.1186/s12879-021-06676-6.

## Background

Tuberculosis (TB) is still a major public health concern especially in low resources settings. In 2019, among the 10 million of people affected, there were 1.4 million deaths [1]. Since decades, the diagnosis of TB is standardized and based on World Health Organization (WHO)’ recommendations, evolved over time to improve diagnostic services. In 2003, for screening in TB-endemic countries, WHO recommended for the diagnosis of pulmonary TB, the use of microscopic examination of two or three sputum specimens for detection of acid-fast bacilli (AFB) [2]. Indeed, several studies had shown that testing multiple specimens yields an increase in sensitivity compared to culture, the reference method for TB detection. This incremental yield was 11.1% for the second specimen (morning specimen, MS) compared to the first specimen (spot specimen, SS) and only 2–5% for the third specimen [3, 4]. Moreover, the bacterial load of the MS was described to be higher than that of the SS [5–7]. WHO therefore recommended two specimens, including a MS, to diagnose pulmonary TB with microscopy in endemic countries [8, 9]. Subsequently, nucleic acid amplification tests such as the GeneXpert® MTB/RIF (Cepheid, Sunnyvale, USA) have been developed. This new technology showed a higher sensitivity than microscopy, is user-friendly and results are available within 2 h. In addition, it can detect resistance to rifampicin (RIF), the main first-line anti-TB drug [10, 11]. WHO thus recommended in 2013 the use of GeneXpert® MTB/RIF as initial test for rapid and simultaneous detection of TB and RIF resistance in all TB suspected cases [12]. The recommendation supports the use of a single sputum but highlights that multiple specimens may increase the diagnostic sensitivity, yet recognizes resource implications. On the other hand, the preferred specimen between a SS and a MS especially in programmatic conditions in low income countries was not mentioned. Nevertheless, MS is associated with increased sensitivity of microscopy as initial diagnostic test. But to the best of our knowledge there are no studies evaluating in programmatic conditions the possible added value of the MS when GeneXpert® MTB/RIF is used as initial diagnostic test. One study evaluated the sensitivity of GeneXpert® MTB/RIF test on MS versus SS and showed no significant difference. However, the study was restricted to smear-negative patients and the sample size was limited [13]. Furthermore, the possible impact of a screening strategy based on MS collection was not evaluated. In this study, we assessed the contribution of different specimens’ collection strategies for pulmonary TB screening using GeneXpert® MTB/RIF in Cotonou, Benin.

## Methods

### Study design

Between June and September 2018, a prospective cross-sectional study was conducted and included all consecutive patients with presumptive pulmonary TB (new and relapse cases).

### Study setting

The study was carried out at the Centre National Hospitalier de Pneumo-Phtisiologie de Cotonou, the biggest public tertiary hospital specialized in TB in Benin. The hospital is located in the south of the country but also serves as referral center for the whole country. Bacteriological diagnosis of TB in this hospital is performed by the WHO Supranational Reference Laboratory for TB in Cotonou, with a turnaround time of 24 h for the GeneXpert® MTB/RIF. Therefore, patients are asked to return to the clinic the day after the initial visit to obtain their result.

### Specimen collection

After informed consent, patients were asked to provide two sputum specimens: the first one was collected on site (SS) and the second was provided the next morning after collection at home and brought to the laboratory (MS). A phone call was made to the patients who did not bring the MS.

### Specimen processing

Specimens were processed with the GeneXpert®MTB/RIF cartridge according to the manufacturer’s instructions [14]. RIF-resistant results were further confirmed by repeating the test on an additional specimen as recommended for new cases [15]. Once confirmed as RIF-resistant, this specimen was processed for culture and indirect phenotypic drug-susceptibility testing (pDST) on Löwenstein Jensen media [16, 17].

### Statistical analysis

The overall percentage of agreement (OPA), positive percent agreement (PPA) and the negative percent agreement (NPA) with 95% Wilson score confidence intervals were estimated to evaluate the performance of GeneXpert®MTB/RIF using SS and MS. The McNemar test was used to assess the evidence of agreement for GeneXpert®MTB/RIF results obtained from SS and MS. Subsequently, the Cohen’s Kappa coefficient was used to evaluate the concordance between the bacterial load by GeneXpert®MTB/RIF and the type of sputum (SS or MS) [18]. All analyses were performed using Stata version 14.2 [19].

## Results

In total, 886 presumptive TB patients were included in the study of whom 480 (54.2%) were male, 859 (97.0%) had never suffered from TB while 27 (3.0%) had a history of TB (Additional file 1, Table 1). There was no evidence of difference in the distribution of age and history of TB across those who provided both SS and MS and those who only provided SS (p = 0.65 and p = 0.43, respectively). However, women were more likely to return with a MS (p = 0.02).

**Table 1:** Agreement between results from paired spot specimens (SS) and morning specimens (MS) using GeneXpert® MTB/RIF

Spot specimen	Morning specimen			P^a^	OPA (95% CI)^b^	PPA (95% CI)^b^	NPA (95% CI)^b^
	Positive	Negative	Total				
Positive	152	3	155	0.99	99.2 (98.2–99.6)	98.1 (94.5–99.3)	99.5 (98.5–99.8)
Negative	3	579	582				
Total	155	582	737^&^				

*OPA* overall percentage of agreement; *PPA* positive percent agreement; *NPA* negative percent agreement

^&^The agreement analysis included 737 presumptive TB patients who provided both SS and MS

^a^McNemar test

^b^Wilson 95% confidence intervals

Of the 886 patients, 654 (73.8%) returned to provide the MS as requested, while 83 (9.4%) provided the MS after a reminder by phone, and the remaining 149 (16.8%) did not provide it despite the phone call. Thus, 737 provided both SS and MS and 149 provided only SS. Among the 232 patients who did not initially provide the MS, 37 (15.9%; 95% CI 11.2–20.7) had TB detected on the SS.

Agreement was evaluated using paired specimens only, thus in patients who provided both SS and MS. From 737 patients providing both SS and MS, 155 (21.0%) were TB-positive by GeneXpert® MTB/RIF for both samples (Table 1). Using either SS or MS as reference standard, the PPA and NPA were 98.1% (95% CI 94.5–99.3) and 99.5% (95% CI 98.5–99.8), respectively. The overall percentage of agreement was high (99.2%, 95% CI 98.2–99.6) and there was no evidence of disagreement between the two types of specimen (p = 0.99). There were 731 concordant and six discordant results; the bacterial load ranged from ‘’very low to medium’’ in the specimens that tested positive in either specimen type.

Comparing the bacterial load of paired SS and MS the kappa coefficient was 0.35 and suggested poor agreement between SS and MS (Table 2).

**Table 2:** Agreement between bacterial load of the positive specimens using GeneXpert® MTB/RIF on paired spot specimens and morning specimens

Bacterial load from morning specimens	Bacterial load from spot specimens				
	High	Medium	Low	Very low	Total
High	28	24	4	1	57
Medium	6	45	14	2	67
Low	0	7	12	2	21
Very low	0	0	5	2	7
Total	34	76	35	7	152

Kappa coefficient = 0.35

Finally, four RIF resistant (all were new cases) were detected on MS, of which two were also detected on SS (Table 3). The concordant RIF-resistant specimens had a bacterial load ranging from “high” to “low”. The two discordant specimens had a “very low” bacterial load in MS while the bacterial load in SS was “low”. After repeating GeneXpert® MTB/RIF on an additional specimen, the two discordant specimens initially resistant by MS, were found RIF-susceptible with a “low” bacterial load. pDST confirmed all concordant results and RIF-susceptible results for the discordant cases.

**Table 3:** Resistance status to rifampicin by GeneXpert MTB/RIF on paired spot specimens and morning specimens

Morning specimen	Spot specimen		
	Resistant	Susceptible	Total
Resistant	2	2*	4
Susceptible	0	148	148
Total	2	150	152

*Confirmed as RIF-susceptible by repeat GeneXpert MTB/RIF and phenotypic drug-susceptibility testing

## Discussion

Of the included patients, more than a quarter did not provide the MS as per agreement, requiring a reminder by phone, which led to return to the clinic in a third of those who received the reminder. Under programmatic conditions, especially in low resources settings, an algorithm that includes a phone call to such a high number of presumptive patients may be difficult to implement because of economic implications and feasibility in routine conditions. Therefore, with a TB screening strategy based on GeneXpert® MTB/RIF on MS only, these presumptive patients could be lost. Moreover, 15.9% of these “loss to diagnostic follow-up” were diagnosed with TB. The MS-only strategy would be detrimental to global efforts to increase TB notifications. Indeed, giving a specimen at the hospital on the day of arrival probably creates a link between the patient and the hospital and motivates him/her to come back the next day to get the result. This is not the case with the MS-only strategy.

GeneXpert® MTB/RIF showed a similar positivity rate for SS and MS with excellent OPA, PPA and NPA greater than 98%, which is consistent with what was previously reported [13].

Although the overall positivity rate on GeneXpert MTB/RIF® was similar, the poor agreement between the bacterial load of two types of samples suggests that the bacterial load of MS is higher than that of SS. This confirms previous studies’ results based on microscopy which noted the superiority of MS over SS in terms of bacterial load [5–7]. Nevertheless, both sample types had the same number of “very low”, so the difference was mainly in the grading of the samples with a higher bacterial load.

The overall high positivity rate observed in this study (21% of presumed TB cases) could be explained by the fact that the study took place in a tertiary hospital for TB. Besides, it cannot be excluded that patients may have arrived late at the hospital in a deteriorated state of health.

The false negative cases noted in each group (SS and MS) could be explained by the intermittent nature of the bacilli released in sputum after cough that has been described in pulmonary TB patients [20]. Also the heterogeneous distribution of bacilli in the same specimen may have had impact, as the processed part may not contain any bacilli.

Among the four RIF-resistant cases recorded, two were false resistant. These discrepancies have been described as strongly associated with a “very low” bacterial load [21]. Indeed, the algorithm used to detect RIF resistance in GeneXpert® MTB/RIF is based on delay or absence of binding of five probes (labelled A–E) that cover the 81-bp RIF-resistance determining region. Thus, factors such as insufficient DNA in the specimen or silent mutation may lead to a false detection of RIF resistance [22]. To overcome these limitations, the GeneXpert® MTB/RIF Ultra has been introduced. In this cartridge, the detection of RIF resistance is based on the interpretation of the melting curves of molecular probes [23]. However, recent studies showed that for detection of RIF resistance, GeneXpert Ultra and GeneXpert® MTB/RIF had similar sensitivity and specificity [24, 25], and post-implementation population based studies are still to be performed. Another cause for the discrepant results could be the presence of minority *rpoB* mutant populations in the processed samples that were less fit to grow in culture, as was previously described for ‘borderline’ *rpoB* mutants [26]. While such mutations may be more frequently presenting as ‘heteroresistance’, which can easily be missed in GeneXpert (both MTB/RIF and Ultra requiring > 50% mutant population to recognize RIF resistance), they are not associated with lower bacterial burden than common *rpoB* mutants. Needless to say, the interpretation of RIF resistance in the case of a very low bacterial load should be done with great care and should follow published guidelines [15].

## Conclusion

In conclusion, both SS and MS yielded similar rates of TB diagnosis by GeneXpert® MTB/RIF in presumptive patients, but a screening strategy based on MS may lead to loss to follow up of patients. SS is therefore suitable as initial sample for TB diagnosis by GeneXpert® MTB/RIF.

## Supplementary Information

**Additional file 1: Table 1.** Participants’ characteristics by number of specimens provided.

## Data Availability

The datasets used during the current study can be obtained from the corresponding author on request.

## Abbreviations

MS: Morning specimen
NPA: Negative percent agreement
OPA: Overall percentage of agreement
SS: Spot specimen
pDST: Phenotypic drug susceptibility testing
PPA: Positive percent agreement
TB: Tuberculosis
