# Supplementary material for: Spot specimen testing with GeneXpert MTB/RIF results compared to morning specimen in a programmatic setting in Cotonou, Benin
Source: BMC Infect Dis. 2021 Sep 20;21:979. doi: 10.1186/s12879-021-06676-6 (PMC8454072; doi:10.1186/s12879-021-06676-6)
Supplement: Supplementary file 1 — Additional file 1: Table 1. Participants’ characteristics by number of specimens provided. [file 12879_2021_6676_MOESM1_ESM.docx]

**Additional file**

Additional file 1, Table 1, Participants’ characteristics by number of specimens provided

| **Characteristics** | **All enrolled participants**  **(n=886)** | **Provided spot and morning specimens**  **(n=737)** | **Provided spot specimen only**  **(n=149)** | **P** |
| --- | --- | --- | --- | --- |
| Female gender n (%) | 406 (45.8) | 351 (47.6) | 55 (36.9) | 0.02^a^ |
| Age group in years, n (%) |  |  |  | 0.65^a^ |
| Below 15 | 62 (7.0)) | 15 (6.9) | 11 (7.4) |  |
| 15-29 | 228 (25.7) | 185 (25.1) | 43 (28.9) |  |
| 30-44 | 308 (34.8) | 257 (34.9) | 51 (34.2) |  |
| 45-59 | 186 (21.0) | 161 (21.8) | 25 (16.8) |  |
| 60 and above | 102 (11.5) | 83 (11.3) | 19 (12.7) |  |
| History of TB, n (%) |  |  |  | 0.43^b^ |
| New | 859 (97.0) | 716 (97.2) | 143 (96.0) |  |
| Relapse | 27 (3.0) | 21 (2.8) | 6 (4.0) |  |

TB. Tuberculosis. ^a^ Chi square test. ^b^ Fisher’s exact test
